# Multimodal integration of neuroimaging, transcriptomics and single-cell analysis reveals molecular correlates linking osteoporosis to brain abnormalities

**DOI:** 10.3389/fimmu.2026.1817475

**Published:** 2026-05-18

**Authors:** Min Fang, Nan Li, Wenyue Xu, Yuan Xue, Rui Wang, Jiaming Zhou

**Affiliations:** 1 Department of Orthopedic Surgery, Tianjin Medical University General Hospital, Tianjin, China; 2 Department of Orthopedic Surgery, Tianjin Hospital, Tianjin, China; 3 Department of Ultrasound, Tianjin Hexi District Liulin Hospital, Tianjin, China; 4 Department of Orthopedic Surgery, Affiliated Hospital of Hebei University, Baoding, China

**Keywords:** Allen Brain, astrocyte, cognitive decline, fMRI, osteoporosis

## Abstract

**Background:**

Osteoporosis (OP) and cognitive decline are highly prevalent comorbidities; however, the molecular mechanisms linking them remain unclear. We adopted a multimodal integrative strategy combining neuroimaging, transcriptomics, single-cell analysis, and in vivo validation to elucidate potential mechanisms.

**Methods:**

Fifty-six patients and fifty-three healthy controls underwent resting-state functional MRI (fMRI) to assess regional homogeneity (ReHo) and amplitude of low-frequency fluctuation (ALFF). Transcriptomic data from the Allen Human Brain Atlas (AHBA), single-nucleus RNA sequencing (snRNA-seq) of the human hippocampus, and validation using ovariectomized (OVX) mouse models were integrated.

**Results:**

fMRI revealed significant ALFF/ReHo alterations in the hippocampus, prefrontal cortex, and posterior cingulate cortex of OP patients, with the left hippocampal ALFF mediating the association between bone mineral density (BMD) and Montreal Cognitive Assessment (MoCA) scores. Spatial correlation analyses have linked these brain functional changes to neurotransmitter receptors (5-HT1a, D1, GABAa, etc.) and genes enriched in synaptic function, neurogenesis, and dopaminergic signaling. snRNA-seq identified the caudal hippocampus as a key region, with astrocytes enriched in OP-associated Gene Program 5 (involving NR4A3 and KCNIP1) and functional pathways such as glutamatergic synapses and calcium signaling. OVX mice showed bone loss, spatial learning/memory impairment, hippocampal astrocyte abnormalities, and upregulation of GFAP, RGS7, and RGS6 proteins.

**Conclusion:**

Our multimodal study establishes a molecular framework for the bone-brain axis, highlighting astrocytes and synaptic signaling as potential targets for the dual protection of bone and brain health.

## Introduction

With global population aging at an unprecedented rate, age-related comorbidities have emerged as major public health challenges, among which osteoporosis (OP) (1) and cognitive dysfunction (2) stand out for their high prevalence and devastating impacts. OP, defined as a systemic skeletal disorder characterized by reduced bone mass, deteriorated bone microarchitecture, and increased fracture risk, affects > 30% of the elderly population worldwide (3). A growing body of epidemiological and imaging evidence has documented a robust association between low bone mineral density (BMD) and cognitive decline in patients with OP (4, 5). Longitudinal cohort studies have shown that OP patients are at a higher risk of developing mild cognitive impairment (MCI) (5) and a 2-fold higher risk of Alzheimer’s disease (AD) than age-matched individuals with normal bone mass (6). Cross-sectional neuroimaging studies have further corroborated this link by identifying correlations between reduced BMD and structural atrophy in the hippocampus, prefrontal cortex, and temporal lobe, brain regions critically involved in memory and executive function, and functional connectivity disruptions in the default mode network and salience network (7). Despite these compelling observations, the field is still constrained by phenotypic associations, as the underlying molecular mechanisms linking OP to brain abnormalities remain largely unknown.

Several preclinical studies have shown that bone-secreted proteins impact the brain and therefore affect cognition (8, 9). Current research on the OP-brain axis suffers from several major limitations that hinder translational progress. First, the underlying molecular mechanisms remain elusive and poorly characterized. Most existing studies have focused on descriptive associations between BMD metrics and neuroimaging phenotypes but have failed to dissect the cellular and molecular cascades that associated with the crosstalk between skeletal aging and cerebral dysfunction (10, 11). Although hypotheses such as systemic inflammation, oxidative stress, and shared endocrine regulatory pathways (e.g., vitamin D and parathyroid hormone dysregulation) have been proposed, direct evidence linking these factors to specific brain region abnormalities in OP is lacking. Second, single-modality research predominates, leading to an incomplete understanding of the multidimensional pathological processes. Traditional studies either rely solely on neuroimaging (10) to describe structural/functional changes or use bulk transcriptomics to analyze gene expression in isolated tissues (12), ignoring the spatial correspondence between regional brain abnormalities and molecular alterations. This disconnection between macroscopic imaging phenotypes and microscopic molecular events makes it difficult to identify key genes or pathways that drive region-specific brain changes in OP patients. Third, insufficient cellular resolution and lack of *in vivo* validation further constrain mechanistic understanding. Bulk RNA sequencing analysis overlooked cell type-specific molecular signatures and failed to distinguish distinct cell subtypes in the brain (13). This limitation not only obscures cell-type-specific gene expression alterations, but also impedes the identification of “disease-critical gene programs” a coordinated molecular signature that defines cell identity, and pathological processes (14). Furthermore, most OP brain neuroimaging studies are macroscale, and the absence of animal experiments precludes the validation of potential molecular targets.

To address these critical gaps, we propose an innovative multimodal integrative strategy that combines neuroimaging, transcriptomics, single-cell analysis, and *in vivo* validation to elucidate the molecular basis of brain abnormalities in OP. The core advantage of our approach lies in the systematic integration of multidimensional data, which enables the translation of macroscopic imaging phenotypes into microscopic molecular insights. First, we leverage neuroimaging-transcriptome association analysis by spatially correlating group-difference statistical maps derived from brain functional magnetic resonance imaging (fMRI) with the spatial expression patterns of the Allen Human Brain Atlas (15, 16). This technique allowed us to identify genes whose spatial expression profiles significantly overlapped with brain regions showing OP-related structural or functional abnormalities, thereby establishing a direct link between regional brain dysfunction and molecular signatures. Second, we incorporated single-cell RNA sequencing (scRNA-seq) data from human brain tissues and adopted a validated analysis workflow. By applying the Cell-type-specific Expression (CELLEX) algorithm (14), we quantified the region- and cell-type-specific expression profiles and identified OP-associated gene programs within the brain. This approach overcomes the limitations of bulk sequencing by resolving cellular heterogeneity, identifying disease-relevant cell subtypes, and uncovering cell-type-specific gene expression changes that may associated with OP-brain interaction. Third, we validated our human-derived findings using a mouse model of OP, which recapitulates bone and brain changes observed in humans. Through longitudinal monitoring of bone mass (via micro-CT) and targeted molecular manipulation, we established an association between the identified molecular pathways and brain abnormalities, thereby providing a solid preclinical foundation for translational research.

In summary, the current understanding of OP-related brain abnormalities is constrained by a lack of mechanistic insights, single-modality bias, and insufficient validation. Our multimodal strategy, integrating neuroimaging-transcriptome association, single-cell resolution analysis, and animal experimental validation, addresses these limitations by bridging macroscopic and microscopic observations. This study aimed to identify the molecular targets that connect OP to brain dysfunction, ultimately providing a theoretical basis for the development of novel therapeutic interventions to simultaneously protect bone and brain health in the elderly population.

## Materials and methods

### Subjects and cognitive assessment

Prior to data collection, the sample size for the present study was established by referencing earlier research (17). Accordingly, 56 patients with osteoporosis (OP) and 53 healthy controls were recruited; all participants were advised to maintain their regular daily routines throughout the study period. This research was approved by the local ethics committee, and all participants provided written informed consent prior to their involvement. In the present study, the diagnostic criteria for osteoporosis (OP) adhered to the guidelines established by the World Health Organization (WHO) (**Supplementary Methods**-Inclusion and exclusion criteria).

Before MRI scanning, cognitive function was evaluated in both the patient groups and healthy control subjects. MoCA (18) is a widely recognized tool for detecting Mild Cognitive Impairment (MCI) and is known for its high sensitivity. An MoCA score of 26 or above indicates normal cognitive function. To account for educational differences, one extra point was given to participants with fewer than 12 years of formal schooling. All MoCA evaluations were performed in a quiet setting by trained assessors to ensure consistency and accuracy.

### Neuroimaging-transcriptional association analyses

After data preprocessing (**Supplementary Methods**-fMRI data acquisition and preprocessing), Regional Homogeneity (ReHo) and amplitude of low-frequency fluctuations (ALFF) were computed at the voxel level. The ALFF and ReHo analyses were performed using the DPARSF toolbox. These two metrics capture distinct and complementary aspects of resting-state brain function. Therefore, combining ALFF and ReHo analyses allowed us to obtain a more comprehensive and multidimensional assessment of resting-state brain functional alterations in patients with osteoporosis, avoiding the one-sidedness of using a single metric. To explore the potential brain mechanisms linked to OP, a voxel-wise two-sample t-test was conducted. Age, sex, years of education, and total intracranial volume (TIV) were incorporated as covariates to account for their possible influence on the ReHo and ALFF. This analysis was carried out within a gray matter mask, restricting the inquiry to study-relevant brain regions. Family wise error (FWE) correction was implemented at the cluster level to address multiple comparisons. With this correction, a corrected p-value < 0.05 was considered statistically significant, and the voxel-wise significance threshold was set to p ≤ 0.001. To contextualize the observed functional alterations, we performed surface-based spatial mapping of all statistically significant ALFF and ReHo vertices onto the Yeo 2011 7-network atlas in the standard fsaverage space. Vertex-wise overlap analysis was conducted to quantify the distribution of significant functional changes across the seven canonical functional networks. The percentage of overlap was defined as the number of vertices showing significant between-group differences that overlapped with a specific network from the Yeo 2011 7-network atlas (19), divided by the total number of vertices showing significant between-group differences.

Transcriptomic data from the Allen Human Brain Atlas (AHBA) were integrated using a standardized processing framework via the abagen toolbox (https://github.com/netneurolab/abagen) following established neuro-genomic protocols (20) (**Supplementary Methods**-AHBA preprocessing). Associations between transcriptional profiles and neuroimaging markers of abnormal brain function in OP were investigated using Spearman’s ρ correlation analysis, comparing transcriptional data with resting-state fMRI contrast maps (OP vs. control groups). To handle multiple comparisons in genome-wide univariate screening (n=10, 185 genes), a dual-threshold framework was adopted: 1) spatial autocorrelation-adjusted permutation testing (10, 000 Monte Carlo iterations to generate empirical null distributions) with a cluster-forming threshold of p<0.05; 2) family wise error correction via Bonferroni adjustment (α=4.91×10^-6^). The gene set selected for functional annotation consisted of overlapping genes from both the ALFF and ReHo analyses. Significant genomic loci were subjected to multi-level functional annotation using ToppGene Suite (21) (https://toppgene.cchmc.org), which identifies enriched biological processes through hypergeometric testing with Benjamini-Hochberg FDR correction (q<0.05). This multimodal analytical workflow provided strict control over both spatial and genomic confounds while linking molecular pathways to neurofunctional phenotypes.

### Neuroimaging-neurotransmitter/metabolism association analyses

The JuSpace toolbox (v1.5) (22) was used to assess whether the spatial patterns of ALFF/ReHo alterations in OP patients versus healthy controls (HCs) were associated with the distribution maps of specific neurotransmitter receptors or transporters. Details could be found in **Supplementary Methods**-Neuroimaging-neurotransmitter/metabolism Association Analyses.

### Single-nucleus RNA sequencing data analyses

Single-nucleus RNA sequencing (snRNA-seq) data from the human brain were acquired in this study. Transcriptomic datasets were retrieved from Siletti et al. (2023) (23) and the CELLxGENE repository (https://cellxgene.cziscience.com). To analyze the snRNA-seq data, we first combined datasets from all brain regions into a single unified dataset (**Supplementary Figure S2**). Preprocessing steps could be found in **Supplementary Methods**-Single-nucleus RNA sequencing (snRNA-seq) data preprocessing. The cell types were annotated based on previously published canonical marker genes. For instance, astrocytes were identified by the marker genes AQP4, GFAP, and GJA1; Schwann cells by MPZ and S100B; oligodendrocytes by MOG and MOBP; and oligodendrocyte precursor cells (OPCs) by VCAN. Other cell populations such as CNS macrophages, leukocytes, fibroblasts, and neurons were annotated using well-validated marker genes in the same manner.

**Figure 1:**
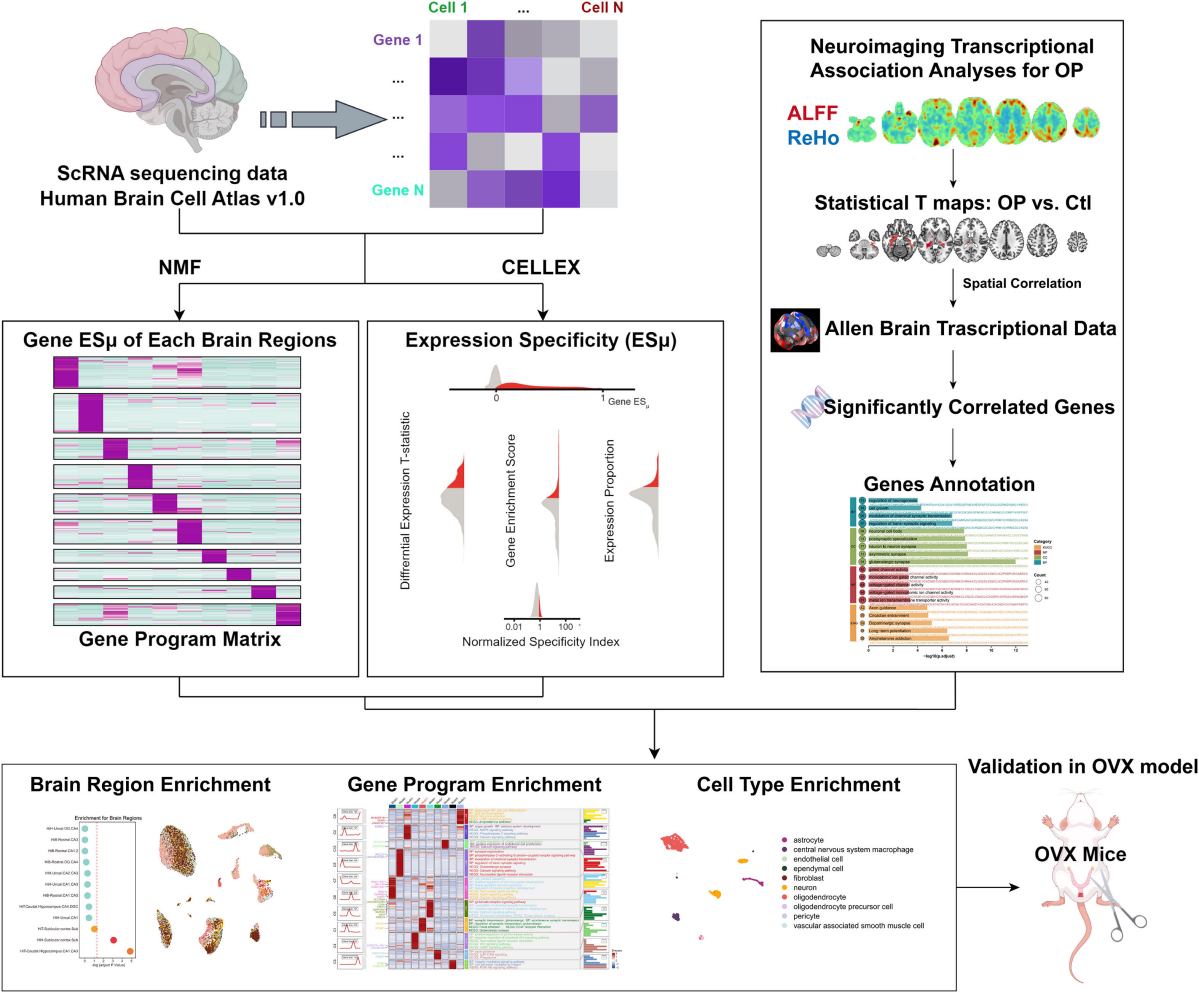
Schematic workflow of integrative analyses linking brain functional alterations, gene expression, and cell types in osteoporosis (OP). Upper left: Single - cell RNA (scRNA) sequencing data from the Human Brain Cell Atlas v1.0 are processed using non - negative matrix factorization (NMF) and CELLEX to derive gene expression specificity (ESμ) and gene program matrices across brain regions. Upper right: Neuroimaging transcriptional association analyses for OP involve correlating statistical T maps of amplitude of low - frequency fluctuation (ALFF) and regional homogeneity (ReHo) (OP vs. controls [Ctl]) with Allen Brain Transcriptional Data to identify significantly correlated genes, followed by gene annotation. Lower panel: Enrichment analyses include brain region enrichment, gene program enrichment, and cell type enrichment. Additionally, validation is performed using an ovariectomized (OVX) mouse model.

To assess the brain region specificity of the gene set identified from neuroimaging-transcriptome association analyses, we used the Cell-type-specific Expression (CELLEX) algorithm (Python module) to compute region-specific expression profiles from the snRNA-seq data (14). The analytical workflow is illustrated in **Figure 1**. CELLEX combines differential expression T-statistics, gene enrichment scores, and expression proportions to measure region-specific gene expression levels. We then conducted an enrichment analysis by intersecting the significantly associated gene sets with region-specific expression profiles. A hypergeometric test was applied to evaluate the statistical significance of gene enrichment in each brain region, with FDR < 0.05, which was used as the cut-off for defining brain regions enriched with the target gene set. Annotations for these enriched brain regions were enhanced by referring to previously published canonical brain region markers and anatomical classification.

Non-negative matrix factorization (NMF) was implemented on the batch-effect-corrected and standardized snRNA-seq expression matrix (via the NMF R package v0.23.0) to break down the transcriptomic data into latent gene programs. The optimal number of gene programs was ascertained using cophenetic correlation coefficients and silhouette scores to guarantee biological significance. Genes within each program were sorted according to their contribution weights and the top 200 high-weight genes per program were chosen for functional annotation. Functional enrichment analysis was carried out using the Kyoto Encyclopedia of Genes and Genomes (KEGG) and Gene Ontology (GO) databases (concentrating on biological processes), with FDR < 0.05 regarded as statistically significant. This step sought to characterize the biological pathways and functional roles of each identified gene.

Finally, we integrated two gene subsets to define the core gene set for protein-protein interaction (PPI) analysis: (1) the neuroimaging-transcriptome associated gene set (generated in the previous neuroimaging-transcriptome association analysis) and (2) the top 200 high-weight genes from the identified gene program. The intersection gene set was uploaded to the STRING database (v11.5, https://string-db.org/), a comprehensive resource for predicting and annotating protein-protein interactions, with Homo sapiens set as the target species. To filter high-confidence interactions, we applied a medium confidence threshold (combined score ≥ 0.4), which is a widely accepted cutoff for balancing sensitivity and specificity in PPI network construction. To explore the biological relevance of the PPI network, we first identified the functional modules (clusters) within the network using the MCODE plugin (v2.0.0) in Cytoscape. MCODE uses a density-based algorithm to detect tightly connected sub-graphs. Each identified module was then subjected to functional enrichment analysis using the clusterProfiler R package (v4.6.0), with enrichment against the KEGG pathway and GO biological process databases. Statistical significance was defined as FDR < 0.05, and only enriched terms with ≥ 3 genes were retained to ensure biological significance.

## Results

### Demographic and clinical assessment

To ensure baseline comparability between the OP and control groups, we compared age, sex, educational level, head motion during scanning, and Montreal Cognitive Assessment (MoCA) scores between the two groups. No significant between-group differences were observed in age, sex, educational level, or head motion during the scanning. The detailed characteristics are presented in **Supplementary Table S1**.

### Regional brain abnormalities in patients with OP

**Figure 2:**
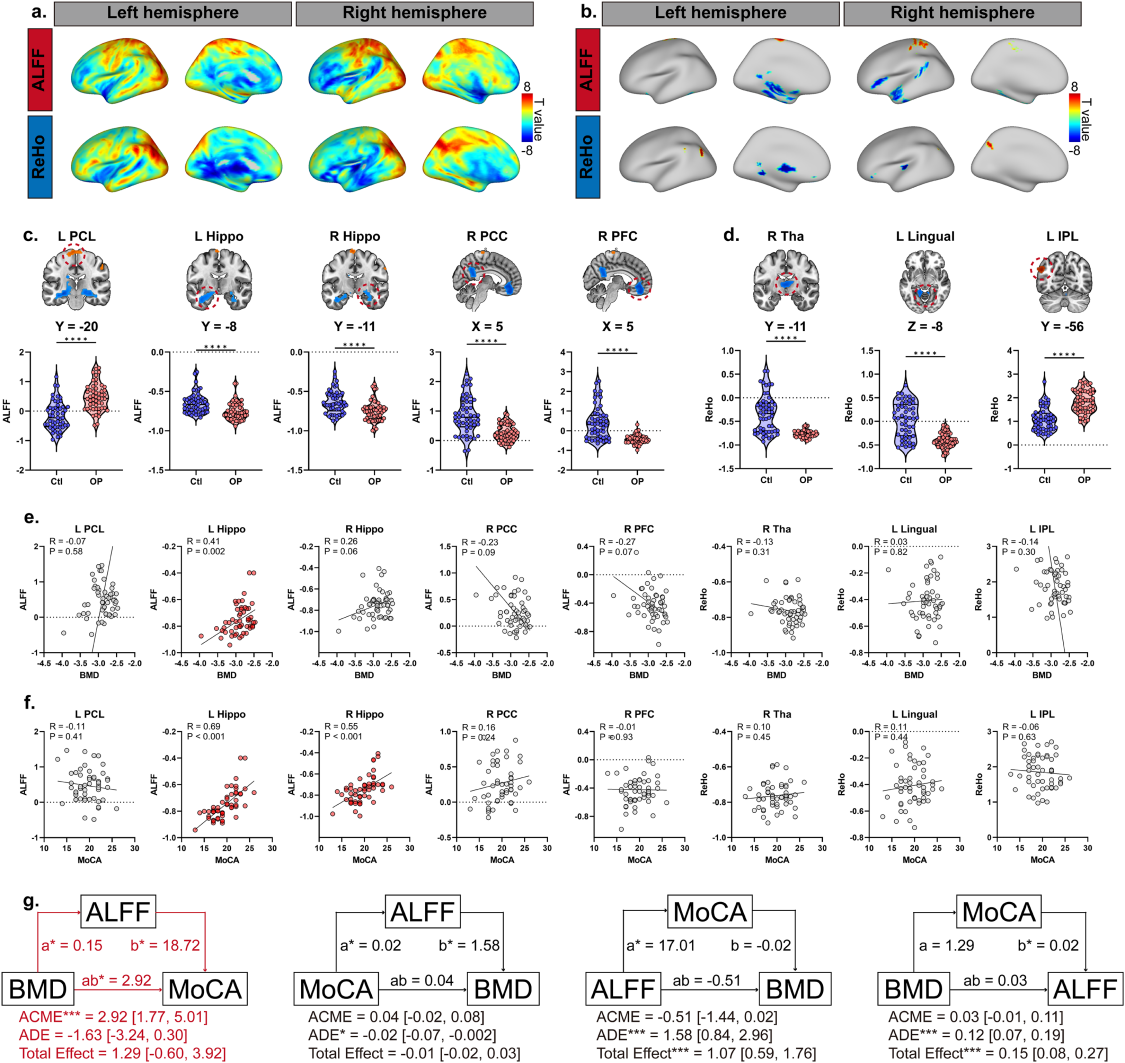
Brain regional abnormalities in osteoporosis (OP) patients compared to healthy controls. Panel **(A)** Spatial distribution of group differences in amplitude of low - frequency fluctuation (ALFF) and regional homogeneity (ReHo) between OP patients and controls, presented for the left and right hemispheres. Panel **(B)** Brain regions showing significant group differences in ALFF and ReHo, visualized for the left and right hemispheres. Panel **(C)** Violin plots and anatomical locations of brain regions with significant group differences in ALFF, including left paracentral lobule (L PCL), left hippocampus (L Hippo), right hippocampus (R Hippo), right posterior cingulate cortex (R PCC), and right prefrontal cortex (R PFC). Panel **(D)** Violin plots and anatomical locations of brain regions with significant group differences in ReHo, including right thalamus (R Tha), left lingual gyrus (L Lingual), and left inferior parietal lobule (L IPL). Panel **(E)** Scatter plots depicting the correlation between bone mineral density (BMD) and ALFF in brain regions with significant group differences in OP patients. Panel **(F)** Scatter plots depicting the correlation between Montreal Cognitive Assessment (MoCA) score and ALFF in brain regions with significant group differences in OP patients. Panel **(G)** Mediation analyses illustrating the path associations among BMD, MoCA, and ALFF of the left hippocampus.

The spatial distributions of the group differences in the ALFF and ReHo are illustrated in **Figure 2A**. Notably, both metrics exhibit a comparable spatial pattern of group differences. Relative to controls, patients with OP demonstrated significantly decreased ALFF in the bilateral hippocampus (Hippo), right posterior cingulate cortex (PCC), and right prefrontal cortex (PFC), whereas ALFF was significantly increased in the left paracentral lobule (PCL) (**Figures 2B, C**). OP patients showed reduced ReHo values in the right thalamus (Tha) and left lingual gyrus, while ReHo was elevated in the left inferior parietal lobule (IPL) (**Figures 2B, D**). Spatial mapping of these significant functional alterations onto the Yeo 2011 7-network atlas revealed a prominent network-specific pattern rather than global brain dysfunction, suggesting both ALFF and ReHo alterations were concentrated in the default mode network (DMN) and the Somatomotor network (**Supplementary Figure S1**).

Pearson correlation analyses were conducted to explore the associations between altered brain functional activity, bone mineral density (BMD), and cognitive performance (assessed using the Montreal Cognitive Assessment [MoCA]). In OP patients, a significant positive correlation was observed between ALFF in the left Hippo and BMD (R = 0.41, p = 0.002, **Figure 2E**). No significant correlations were found between BMD and ALFF/ReHo in other brain regions. Significant positive associations were identified between MoCA and ALFF in the left Hippo (R = 0.69, p < 0.001, **Figure 2F**) and right Hippo (R = 0.55, p < 0.001, **Figure 2F**). No significant associations were detected between MoCA and ALFF/ReHo in other brain regions.

Given the pairwise associations among BMD, left Hippo ALFF, and MoCA in patients with OP, we further investigated the path relationships among these variables using mediation analyses. Four potential models were tested (**Figure 2G**): (1) BMD as the independent variable, left Hippo ALFF as the mediator, and MoCA as the outcome; (2) MoCA as the independent variable, left Hippo ALFF as the mediator, and BMD as the outcome; (3) left Hippo ALFF as the independent variable, MoCA as the mediator, and BMD as the outcome; and (4) BMD as the independent variable, MoCA as the mediator, and left Hippo ALFF as the outcome. Our findings indicated that only Model 1 was significant, with a significant average causal mediation effect (ACME) of 2.92 (95% CI = [1.77, 5.01], p < 0.001), whereas the total effect was 1.29 (95% CI = [-0.60, 3.92], p > 0.05). These results suggest that an altered ALFF in the left hippocampus may contribute to the association between osteoporosis and cognitive ability.

### Neurotransmitters and transcriptome association with brain regional abnormalities in patients with OP

**Figure 3:**
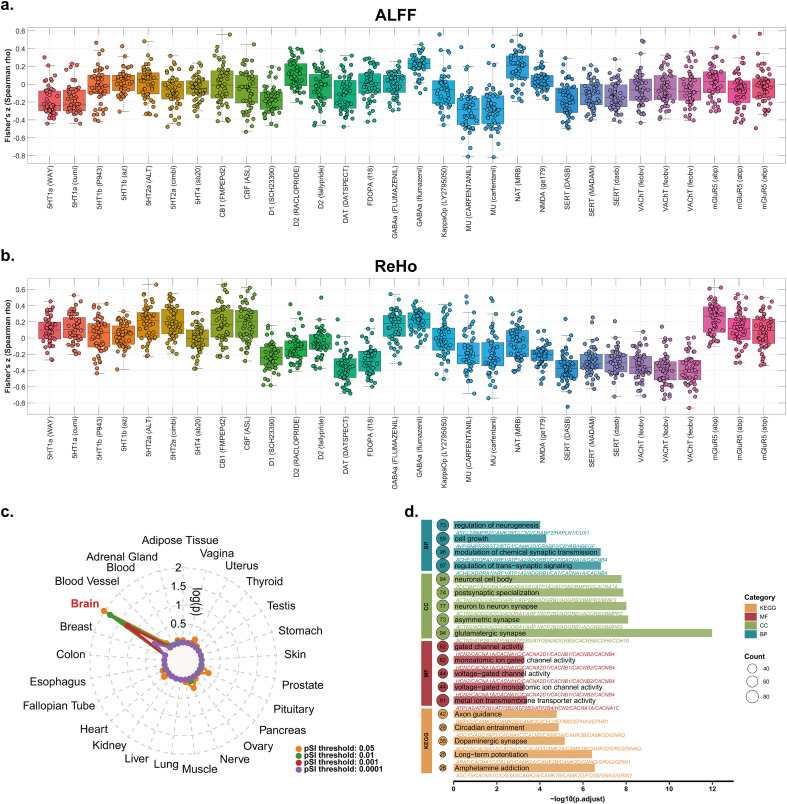
Associations between OP - related brain functional alterations, neurotransmitter receptors, and gene expression. Panel **(A)** Scatter plots with box - and - whisker diagrams illustrating Fischer’s z - transformed Spearman correlation coefficients between voxel - based ALFF differences in OP patients and the spatial distribution of various neurotransmitter receptors. Panel **(B)** Scatter plots with box - and - whisker diagrams illustrating Fischer’s z - transformed Spearman correlation coefficients between voxel - based ReHo differences in OP patients and the spatial distribution of various neurotransmitter receptors. Panel **(C)** Tissue - specific enrichment analysis of genes associated with OP - related regional brain functional abnormalities, showing prominent enrichment in the brain across multiple pSI thresholds. Panel **(D)** Functional enrichment analysis of the intersecting gene set, depicting enriched terms in Kyoto Encyclopedia of Genes and Genomes (KEGG) pathways and Gene Ontology (GO) categories, including biological process (BP), cellular component (CC), and molecular function (MF).

Compared with controls, OP-related voxel-based ALFF changes were significantly associated with the spatial distribution of 5 - HT1a receptors (WAY: R = −0.19, q = 0.01 FDR - corrected; cumi: R = −0.17, q = 0.006 FDR - corrected), D1 receptors (R = −0.17, q = 0.004 FDR - corrected), D2 receptors (R = 0.11, q = 0.01 FDR - corrected), dopamine transporter (DAT) receptors (R = −0.11, q = 0.02 FDR - corrected), GABA_a_ receptors (flumazenil: R = 0.22, q = 0.003 FDR - corrected), μ opioid (MU) receptors (CARFENTANIL: R = −0.29, q = 0.002 FDR - corrected; carfentanil: R = −0.27, q = 0.002 FDR - corrected), and serotonin transporter (SERT) receptors (DASB: R = −0.17, q = 0.002 FDR - corrected; MADAM: R = −0.12, q = 0.003 FDR - corrected; dasb: R = −0.13, q = 0.004 FDR - corrected). No significant differences in spatial distribution were observed for 5 - HT1b, 5 - HT2a, 5 - HT4, CB1, cerebral blood flow (CBF), FDOPA, KappaOp, NMDA, VAChT, and mGluR5 receptors (all P > 0.05, **Figure 3A**). For voxel-based ReHo alterations in OP patients, compared with controls, significant associations with the spatial distribution of D1 receptors (SCH23390: R = −0.23, q = 0.001 FDR-corrected), DAT (DATSPECT: R = −0.36, q = 0.002 FDR-corrected), FDOPA (f18: R = −0.27, q = 0.002 FDR-corrected), VAChT (feobv: R = −0.39, q = 0.002 FDR-corrected), and mGluR5 receptors (abp: R = 0.23, q = 0.002 FDR-corrected) were observed. No significant differences in spatial distribution were detected for 5 - HT1a, 5 - HT1b, 5 - HT2a, 5 - HT4, CB1, CBF, KappaOp, NMDA, MU, NAT, SERT, or other receptors (all p > 0.05, **Figure 3B**).

We correlated the statistical T-maps of ALFF and ReHo differences between OP patients and controls with the spatial patterns of gene expression. After multiple comparison correction, 1, 672 genes showed a significant correlation with the ALFF T maps, and 1, 728 genes were significantly correlated with the ReHo T maps. We selected the intersection of these two gene sets for further functional annotation (n = 1243). Tissue-specific enrichment analysis (**Figure 3C**) showed that across all tested significance (pSI) thresholds (0.05, 0.01, 0.001, and 0.0001), the genes associated with regional functional activity abnormalities in OP patients were prominently enriched in the brain. This indicates robust brain-specific enrichment of genes linked to OP-related functional brain changes. For functional annotation (**Figure 3D**), we performed enrichment analyses across various categories, including Kyoto Encyclopedia of Genes and Genomes (KEGG) pathways and Gene Ontology (GO) terms (encompassing molecular function [MF], cellular component [CC], and biological process [BP]). In the BP category, significant enrichment was observed in the regulation of neurogenesis, modulation of chemical synaptic transmission, and regulation of transsynaptic signaling. Within the CC category, neuronal cell bodies, postsynaptic specialization, and glutamatergic synapses were enriched. In the MF category, enrichment was found in gated channel activity, monoaminergic ion-gated channel activity, and voltage-gated channel activity. Additionally, KEGG pathway analysis revealed enrichment in axon guidance, dopaminergic synapses, long-term potentiation, and amphetamine addiction, among others. Collectively, these results highlight the involvement of genes related to synaptic function, neurogenesis, and neurotransmission pathways in mediating OP-associated regional brain functional alterations.

### Molecular signatures of osteoporosis-associated brain abnormalities in the hippocampus revealed by single-nucleus transcriptomic data

**Figure 4:**
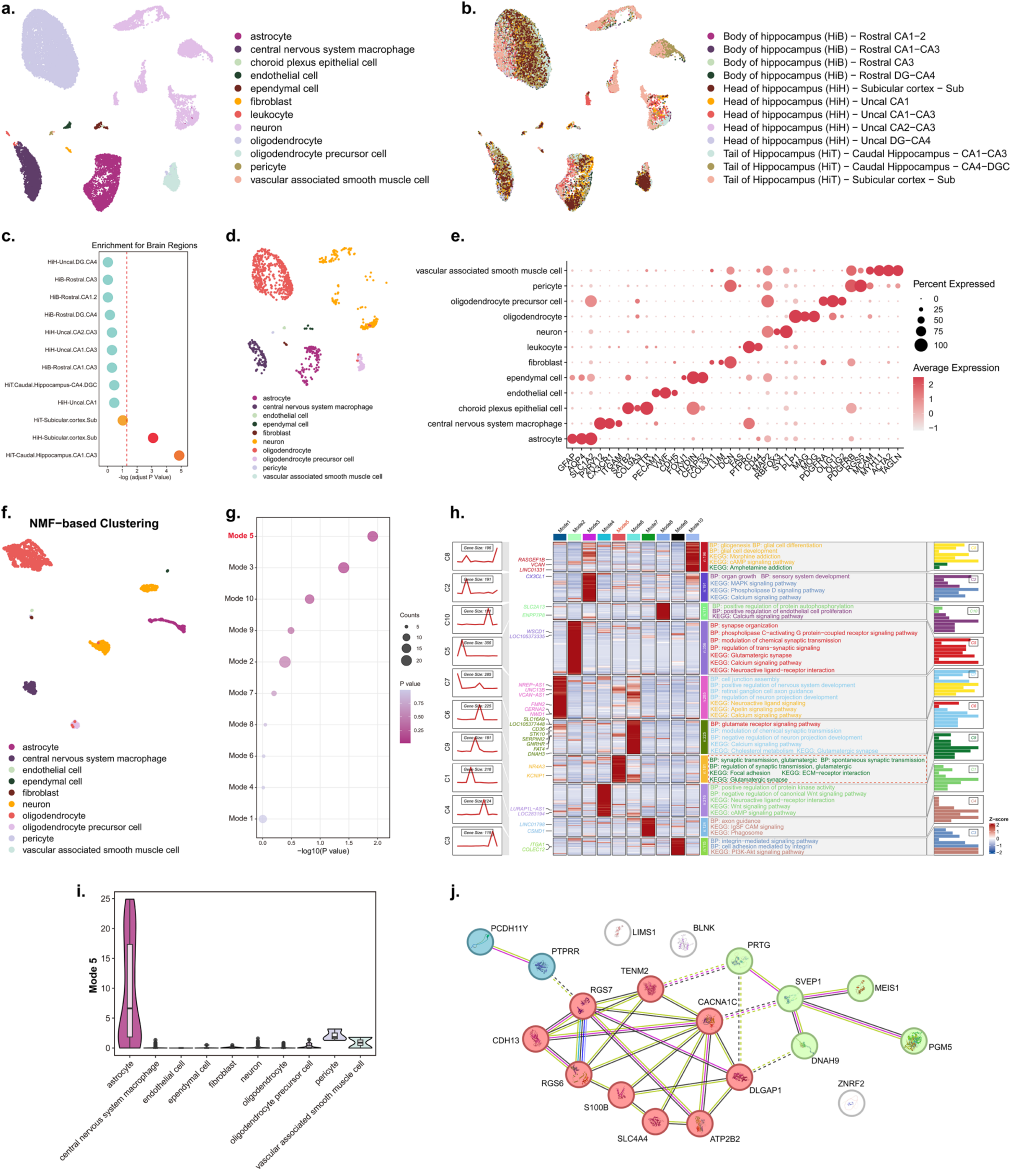
Single-nucleus transcriptomic analysis reveals molecular signatures of osteoporosis-associated brain abnormalities in the hippocampus. Panel **(A)** Uniform manifold approximation and projection (U-map) visualization of major cell types in the human hippocampus, including astrocytes, microglia, neurons, and other glial cells, annotated from snRNA-seq data. Panel **(B)** U-map clustering of hippocampal nuclei by 12 anatomical subregions, illustrating transcriptional heterogeneity across hippocampal areas. Panel **(C)** Enrichment analysis of OP-associated genes in hippocampal subregions, showing significant enrichment in the caudal hippocampal region (HiT-Caudal Hippocampus-CA1-CA3) (adjusted P < 0.05). Panel **(D)** U-map visualization of cell subtypes in the caudal hippocampal region, demonstrating distinct clustering of cell populations. Panel **(E)** Dot plot showing the percent expression and average expression level of marker genes across different cell subtypes in the caudal hippocampus. Panel **(F)** Non-negative matrix factorization (NMF)-based clustering of gene programs from snRNA-seq data of the caudal hippocampus. Panel **(G)** Enrichment of OP-associated genes in NMF-derived Gene Program 5, with significance indicated by -log10(P value). Panel **(H)** Functional enrichment analysis of gene programs, including Gene Ontology (GO) biological processes and Kyoto Encyclopedia of Genes and Genomes (KEGG) pathways, visualized as a heatmap with corresponding enrichment profiles. Panel **(I)** Violin plot depicting the expression distribution of Gene Program 5 across different cell types. Panel **(J)** Protein–protein interaction (PPI) network analysis of intersecting genes from neuroimaging-transcriptomic association and Gene Program 5, illustrating modular architectures involved in synaptic signaling and neurobiological processes.

To elucidate the molecular signatures underlying osteoporosis (OP)-associated brain abnormalities, we analyzed single-nucleus RNA sequencing (snRNA-seq) data from 12 subregions of the human hippocampus, which supports a potential mediating role the association between bone mass and cognitive ability in OP patients. The major cell types, including neurons, oligodendrocytes, astrocytes, and glial cells, were annotated (**Figure 4A**). U-map visualization demonstrated distinct clustering of nuclei by hippocampal subregion, reflecting transcriptional heterogeneity across these anatomical areas (**Figure 4B**).

To characterize the regional enrichment of genes associated with OP-relevant brain abnormalities, we integrated a gene list of OP-associated genes (identified via neuroimaging-transcriptome association analysis) with region-specific expression profiles derived using the CELLEX algorithm. Enrichment analysis revealed that this gene list was significantly enriched in the caudal hippocampal region (HiT-Caudal Hippocampus-CA1-CA3) (adjusted p < 0.05; **Figure 4C**). This region exhibited the highest enrichment score relative to the other 11 hippocampal subregions, with the enrichment score exceeding the predefined significance threshold. These findings suggest that the caudal hippocampus is a key brain region in which OP brain abnormality-associated genes are preferentially expressed, further supporting its central role in the pathogenesis of OP-related brain changes.

We further extracted snRNA-seq profiles from the caudal hippocampal region, and U-map plots revealed a clear segregation of cell subtypes with distinct marker gene expression patterns (**Figures 4D, E**). Non-negative matrix factorization (NMF) decomposed the expression matrix into 10 gene programs, among which Gene Program 5 was significantly enriched in OP-associated genes (**Figures 4F, G**). Key high-weight genes in Program 5 included NR4A3 (e.g., hippocampal development and survival of hippocampal neurons) and KCNIP1 (e.g., neural development and maturation), aligned with known pathophysiological processes of cognitive impairment. GO biological process and KEGG pathway enrichment analyses of the top-ranked genes in each program are shown in **Figure 4H**. Functional annotation showed that Program 5 was significantly associated with multiple neurobiological processes, including the positive regulation of chemical synaptic transmission, glutamatergic synaptic transmission, and regulation of trans-synaptic signaling (BP terms). Correspondingly, Program 5 was enriched in KEGG pathways crucial for neuronal function, such as glutamatergic synapses, neuroactive ligand-receptor interactions, and calcium signaling pathways. Additionally, violin plots demonstrated that program 5 was more highly expressed in astrocytes than in other cell types (**Figure 4I**).

To dissect the protein-level interactions underlying the convergent transcriptional and neuroimaging signatures, we intersected the gene list from the neuroimaging-transcriptomic association analysis with the top-ranked genes in Program 5. Subsequent protein–protein interaction (PPI) network analysis of these intersecting genes revealed a modular architecture (**Figure 4J**, **Supplementary Figure S3**). The network comprises two prominent modules: one densely interconnected around RGS7, RGS6, CACNA1C, and their interactors (e.g., CDH13, S100B, ATP2B2, DLGAP1, SLC4A4, PTPRR, and PCDH11Y), suggesting coordinated roles in synaptic signaling and calcium homeostasis, which is consistent with Program 5 enrichment in neuroactive and calcium-related pathways. The second module consisted of PRTG, SVEPI, MEIS1, PGM5, and DNAH9, forming a relatively distinct subnetwork. Notably, TENM2 and CACNA1C associated with potential crosstalk between the two modules (dashed edges), whereas LIMS1, BLNK, and ZNRF2 exhibit sparse connectivity, implying context-dependent regulatory roles. This modular PPI network underscores that the intersecting genes are organized into functional clusters, with the CACNA1C-centered module likely orchestrating core synaptic processes, and the PRTG/MEIS1-centered module potentially contributing to cell-type specificity or developmental mechanisms in neurobiology.

### Osteoporosis induces hippocampal astrocyte abnormalities and associated protein dysregulation in mice

**Figure 5:**
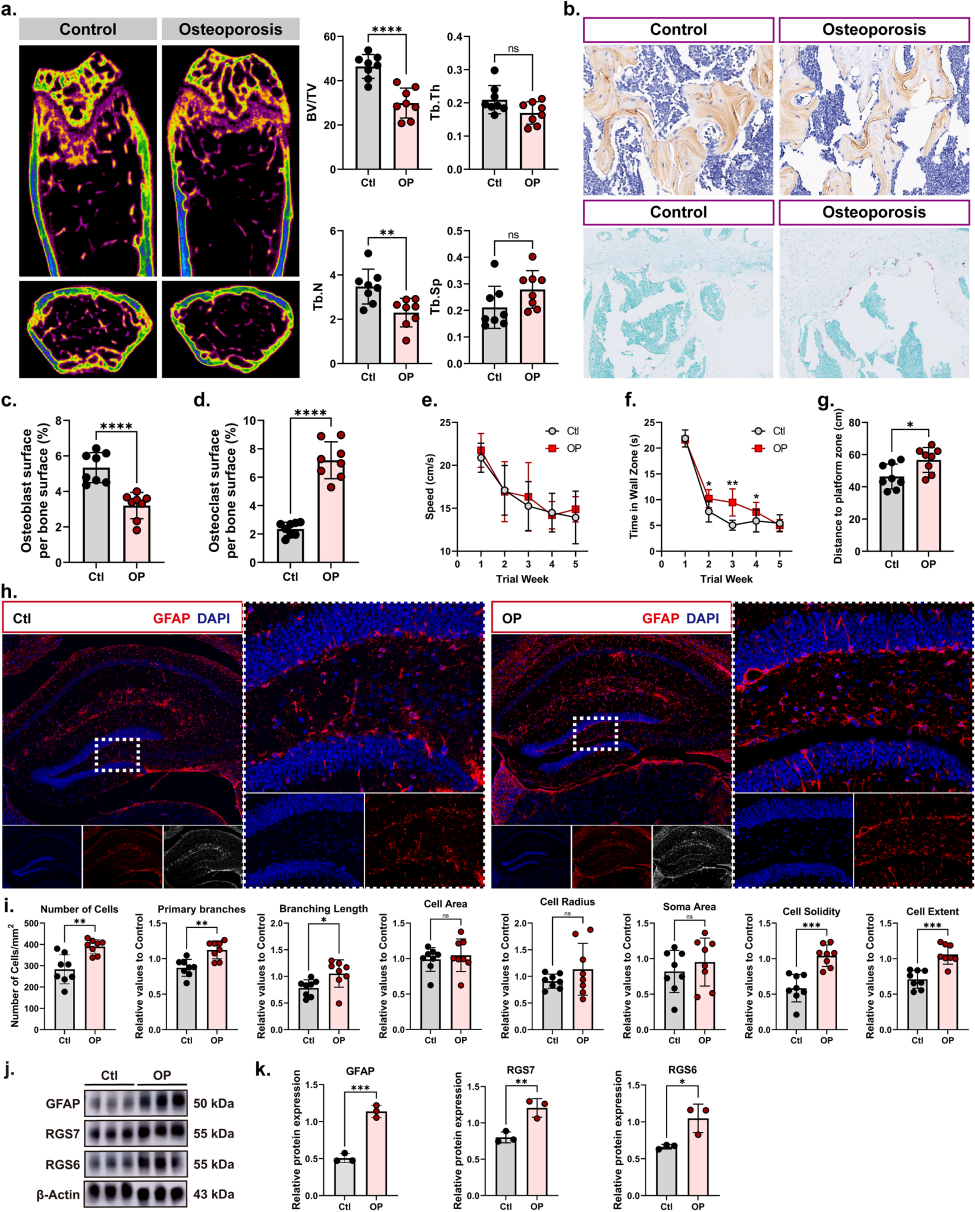
Osteoporosis induces hippocampal astrocyte abnormalities and associated protein dysregulation in mice. Panel **(A)** Micro-CT analysis of trabecular bone in control (Ctl) and osteoporosis (OP) mice, with quantification of bone volume/tissue volume (BV/TV), trabecular number (Tb.N), trabecular thickness (Tb.Th), and trabecular separation (Tb.Sp). ****(p < 0.0001), **(p < 0.01), ns = not significant. Panel **(B)** Histological staining (top: OCN, bottom: TRAP) of bone tissue from Ctl and OP mice, visualizing structural alterations. Panel **(C)** Quantification of osteoblast surface per bone surface in Ctl and OP mice. ****(p < 0.0001). Panel **(D)** Quantification of osteoclast surface per bone surface in Ctl and OP mice. ****(p < 0.0001). Panel **(E–G)** Morris water maze (MWM) assessment of spatial learning and memory in Ctl and OP mice. **(E)** Swimming speed during the 5-day acquisition phase. **(F)** Time spent in the wall zone (a measure of spatial learning, independent of swimming speed). **(G)** Mean distance to the former platform zone in the probe trial. *(p < 0.05\). Panel **(H)**. Representative immunofluorescence images of GFAP (astrocyte marker, red) and DAPI (nuclei, blue) in the hippocampus of Ctl and OP mice, with magnified insets of the dotted boxes. Panel **(I)** Morphological quantification of hippocampal astrocytes in Ctl and OP mice, including cell number, primary branches, branching length, cell area, cell radius, soma area, cell solidity, and cell extent. ****(p < 0.0001), **(p < 0.01), *(p < 0.05). Panel **(J, K)**. Western blot analysis **(J)** and quantification **(K)** of GFAP, RGS7, and RGS6 protein expression in hippocampal tissue from Ctl and OP mice, with β-Actin as control. ****(p < 0.0001), *(p < 0.05).

To validate the single-cell sequencing findings regarding aberrant astrocyte phenotypes and differential expression of their associated proteins in OP, we performed comprehensive multilevel validation analyses. First, micro-CT analysis (**Figure 5A**) showed that OP mice exhibited a significant reduction in bone volume/tissue volume (BV/TV, ****p < 0.0001) and trabecular number (Tb.N, **p < 0.01), whereas trabecular thickness (Tb.Th) and trabecular separation (Tb.Sp) did not differ significantly from the controls (ns), confirming prominent bone mass loss and disruption of trabecular microarchitecture. Histological staining (**Figure 5B**) further visualized these bone structural alterations, and subsequent cellular analyses revealed a marked decrease in osteoblast surface per bone surface (****p < 0.0001, **Figure 5C**) and a significant increase in osteoclast surface per bone surface (****p < 0.0001, **Figure 5D**) in OP mice, reflecting a profound imbalance in bone remodeling homeostasis. To assess whether OP is associated with central nervous system dysfunction, the Morris water maze (MWM) test was conducted 3 months after ovariectomy (OVX) surgery to evaluate spatial learning and memory; during the 5-day acquisition phase, no significant difference in swimming speed was observed between OP and control mice (**Figure 5E**), eliminating speed as a confounding factor—while the swimming speed-independent indicator of spatial learning (time spent in the wall zone) was significantly longer in OP mice (**Figure 5F**), and in the probe trial, OP mice showed a significantly increased mean distance to the former platform zone compared with controls (**Figure 5G**), collectively demonstrating that OP induces significant spatial learning and memory impairment.

Based on our integrated multi-omics analysis, we prioritized GFAP, RGS7, and RGS6 for *in vivo* protein-level validation. RGS7 and RGS6 were among the top 20 highest-weight genes in Gene Program 5, the OP-associated gene program identified by single-nucleus RNA sequencing, and served as core hub nodes in the first functional module of the protein-protein interaction (PPI) network. Additionally, GFAP is a canonical and widely used biomarker of astrocyte activation, and our single-cell analysis demonstrated that Gene Program 5 is predominantly and specifically expressed in hippocampal astrocytes. Therefore, we performed GFAP immunostaining (**Figure 5H**) and morphological quantification (**Figure 5I**), which revealed that OP mice displayed significant changes in multiple astrocyte-related parameters (cell number, primary branch count, branching length, cell area, cell radius, soma area, cell solidity, and cell extent), all of which were statistically significant (****p < 0.0001 or **p < 0.01) relative to control (Ctl) mice, confirming the substantial morphological remodeling of hippocampal astrocytes in OP. Finally, Western blot analysis (**Figures 5J, K**) verified the expression of key molecules identified by single-cell sequencing, showing that OP mice had significantly elevated protein levels of GFAP, RGS7, and RGS6 compared to Ctl mice (****p < 0.0001 or *p < 0.05). Collectively, these multi-level validation data not only confirm the single-cell sequencing-derived insights into astrocyte abnormalities and differential expression of their associated proteins in OP but also establish a multidimensional association between skeletal metabolic dysfunction in OP and structural/functional remodeling of hippocampal astrocytes, linking bone pathology to central nervous system alterations.

## Discussion

In current study, we explored brain functional abnormalities and their molecular correlates underlying OP-cognitive impairment comorbidity, via integration of neuroimaging, neuroimaging-transcriptome association analysis, single-nucleus RNA sequencing and *in vivo* animal models. Our key findings are fourfold: (1) Functional neuroimaging identified significant abnormalities in the hippocampus, left inferior frontal gyrus, and posterior cingulate gyrus of OP patients, correlating with reduced bone mineral density (BMD) and cognitive performance; (2) Neuroimaging-transcriptome analysis identified 1, 243 genes spatially associated with these functional deficits, enriched in synaptic plasticity, dopaminergic synapses, and trans-synaptic transmission regulation; (3) snRNA-seq revealed these OP-related, functionally linked genes are enriched in the hippocampal caudal region, astrocytes, and gene programs tied to glutamatergic synapses/transmission; (4) OVX animal models validated astrocyte abnormalities and upregulation of key proteins from preceding analyses.

This study’s novelty resides in three aspects: First, it is the first to integrate neuroimaging-transcriptome association analysis with snRNA-seq via the CELLEX algorithm, bridging macroscale functional imaging and microscale molecular signatures, overcoming genome-wide association study (GWAS) limitations of reflecting only genetic variation, not functional expression. Second, it links region-specific brain functional abnormalities to protein-level changes, reinforcing the “brain-bone axis” in OP. Third, it identifies cell-type-specific targets (e.g., astrocytes) and key proteins (RGS6, RGS7, GFAP) connecting impaired bone metabolism to cognitive dysfunction, yielding translational implications for dual-targeted therapies.

### Neuroimaging evidence for comorbid osteoporosis and cognitive impairment

Our neuroimaging findings corroborate the emerging concept of the brain-bone axis, revealing that osteoporosis (OP)-associated cognitive decline is associated with functional remodeling of brain regions critical for memory encoding and executive function regulation. In the current study, distinct hemispheric asymmetries in ALFF and ReHo were identified, with the left hemisphere exhibiting more prominent neurofunctional alterations. These changes involve brain regions pivotal to cognitive processing and systemic metabolic homeostasis, including the posterior cingulate lobe (PCL), hippocampus (Hippo), posterior cingulate cortex (PCC), prefrontal cortex (PFC), thalamus (Tha), lingual gyrus, and inferior parietal lobe (IPL). Notably, these regions subserve core functions such as episodic memory consolidation, attentional control, and multisensory integration, which are consistently compromised in cognitive impairments (24). This observation suggests that OP is associated with neurofunctional dysregulation in interconnected neural circuits. We observed significant pairwise associations between bone mineral density (BMD), left hippocampal ALFF values, and MoCA scores, indicating complex interdependencies among skeletal health, hippocampal function, and cognitive performance. Mediation analyses demonstrated that only the model with BMD as the independent variable, left hippocampal ALFF as the mediator, and MoCA as the outcome reached statistical significance, suggesting a potential partial mediating role of left hippocampal ALFF in the association between BMD and cognitive performance. This finding aligns with the “brain-bone axis” hypothesis, wherein bone-derived factors (e.g., osteocalcin and sclerostin) (9, 25) may modulate cerebral function via functional reorganization of specific brain regions (26). However, it is critical to emphasize that our cross-sectional study design cannot establish definitive causal directionality. Alternative directional relationships remain biologically plausible. For example, cognitive decline may precede and contribute to bone loss through reduced physical activity, altered endocrine regulation, or impaired nutritional status; alternatively, hippocampal dysfunction could independently affect both bone metabolism and cognitive performance. The bidirectional nature of the brain-bone axis, where central nervous system signals also regulate skeletal homeostasis, further complicates causal inference. Longitudinal cohort studies and interventional experiments are therefore required to clarify the temporal sequence and causal relationships among these variables.

Additionally, disrupted ALFF in the PCC and PFC, which are core nodes of the default mode network (DMN), may reflect DMN dysfunction, a well-recognized hallmark of early neurodegenerative processes (27). The regional specificity of these neurofunctional alterations, spanning memory, attention, and sensory integration domains, highlights the multifaceted impact of osteoporosis on brain function, which extends beyond isolated cognitive deficits to global neural integration (7, 10). Beyond these observations, OP-related structural changes in the brain share striking similarities with those in other neurodegenerative conditions (28–30). These cross-disease parallels suggest that the brain-bone axis represents a common pathological pathway in age-related degenerative disorders, with neuroimaging serving as a noninvasive and sensitive tool to monitor its dysregulation.

### Transcriptomic and neurotransmitter correlates of brain functional abnormalities in OP

Brain regions subserving the core cognitive domains, including the hippocampus, prefrontal cortex (PFC), and posterior cingulate cortex (PCC), act as nodal hubs for neurogenesis, synaptic plasticity, and large-scale network integration (31). These regions exhibit a convergent molecular profile across multiple functional dimensions. For instance, the transcriptional enrichment of dopaminergic signaling and synaptic plasticity-related genes in these regions underscores their reliance on these pathways for cognitive information processing (31). Transcriptomic alterations driving the dysregulation of these key pathways directly precipitate region-specific neurofunctional abnormalities (e.g., hyperexcitability or hypoactivity) observed in ALFF and ReHo analyses. This link between transcriptional programming and neurofunctional phenotype is associated with a molecular basis for the selective cognitive deficits associated with OP. Furthermore, the overlap between ion channel-related MF (e.g., voltage-gated monatomic ion channel activity) and synaptic CC in these vulnerable regions implies an intrinsic sensitivity to perturbations in both neuronal excitability and synaptic structural integrity (32–34). Such dual sensitivity translates into region-specific functional aberrations, as molecular dysregulation disrupts the spatiotemporal precision of neural activity, which is an essential prerequisite for higher cognitive computations.

For voxel-based ALFF alterations in patients with OP, significant correlations were observed with multiple neurotransmitter systems, including serotonin (5-HT1a), dopamine (D1, D2, dopamine transporter, DAT), γ-aminobutyric acid (GABAa), μ-opioid (MU), and serotonin transporter (SERT) receptors. Notably, the 5-HT1a and dopamine systems are pivotal for emotional regulation (35), cognitive flexibility (36), and synaptic plasticity (37, 38), and dysregulation of these systems may directly underlie ALFF changes in OP, contributing to the cognitive-emotional deficits frequently reported in this patient population. Additionally, GABAa receptors associated with inhibitory neurotransmission (39), while MU receptors regulate reward and stress-resilience pathways (40); their altered associations with ALFF further suggest that OP-associated brain functional abnormalities stem from a disrupted balance between excitatory-inhibitory signaling and impaired stress-adaptive neural circuits. In the context of ReHo alterations reflecting regional functional coherence, significant associations emerged with D1, DAT, FDOPA (a marker of dopamine synthesis), vesicular acetylcholine transporter (VAChT), and metabotropic glutamate receptor 5 (mGluR5). The dopamine system (via D1, DAT, and FDOPA) and cholinergic system (via VAChT) are central to attention allocation (41), memory consolidation (42), and network synchronization (43); their altered spatial correspondence with ReHo likely reflects compromised regional functional integration in OP.

### Single-cell data links brain regions, cell subtypes, and gene programs to brain functional alterations in osteoporosis

The annotation of major cell types (e.g., neurons, oligodendrocytes, astrocytes) and their distinct clustering across hippocampal subregions aligns with established evidence that the hippocampus exhibits profound anatomical and transcriptional heterogeneity, which underpins its functional specialization in learning and memory (44). For instance, studies utilizing snRNA-seq in the human hippocampus have demonstrated that subregion-specific gene expression patterns correlate with differential vulnerability to neurodegenerative processes (45), a concept that extends to OP-associated brain abnormalities. The enrichment of OP-associated genes in the caudal hippocampal subregion is consistent with prior neuroimaging and clinical studies linking OP to structural and functional alterations in the hippocampus (46, 47). This regional specificity suggests that the caudal hippocampus serves as a neuroanatomical hub, where OP-driven molecular events converge to precipitate cognitive deficits.

Within this critical subregion, our snRNA-seq analysis revealed that astrocytes are the predominant cell type expressing program 5, which is enriched in OP-associated genes. This finding is noteworthy given the expanding recognition of astrocytes as active regulators of synaptic function and cognitive processes (48). Astrocytes modulate synaptic transmission via glutamate uptake, gliotransmitter release, and structural support, and their dysfunction has been implicated in a spectrum of cognitive disorders, including AD and vascular dementia (49, 50). In the context of OP, the robust expression of Program 5 in astrocytes points to a novel mechanism wherein OP perturbs astrocyte-associated synaptic regulation, ultimately contributing to cognitive impairment.

Functional annotation of Program 5 highlighted enrichment in processes such as the positive regulation of chemical synaptic transmission, glutamatergic synaptic transmission, and trans-synaptic signaling, all of which are fundamental to cognitive function (51–54). Glutamatergic signaling is central to synaptic plasticity and memory formation, and its dysregulation is a hallmark of cognitive disorders, such as AD (55). The convergence of OP-associated genes within these pathways implies that OP disrupts glutamatergic synaptic homeostasis in the caudal hippocampus, a mechanism that likely underlies the cognitive deficits observed in patients with OP. Moreover, the enrichment of KEGG pathways, such as glutamatergic synapses and calcium signaling pathways, aligns with studies demonstrating that calcium dyshomeostasis in astrocytes impairs synaptic function and contributes to cognitive decline (56), further solidifying the link between astrocyte dysfunction, synaptic pathology, and OP-related cognitive impairment. PPI analyses and *in vivo* validation further revealed that the RGS family members (RGS6, RGS7) modulate G-protein-coupled receptor signaling, which regulates neurotransmitter release and synaptic strength (57), and their involvement in the PPI network disrupts neuromodulatory signaling in OP. Additionally, GFAP, an astrocyte-derived protein, is a biomarker of astrocyte activation and has been linked to synaptic dysfunction in AD (58) and cognitive impairment in patients with OP, reinforcing the role of astrocyte-associated mechanisms.

## Limitations

The current study has several limitations. The sample size of human participants (56 OP patients, 53 controls) was relatively modest, which may limit the generalizability of the neuroimaging-transcriptome association results. The cross-sectional design of human studies prevents the establishment of causal relationships between OP-related molecular changes and brain functional abnormalities; longitudinal follow-up is needed to confirm the temporal dynamics. Single-nucleus RNA sequencing data were derived from postmortem human brain tissues, which may not fully reflect *in vivo* physiological states and could be confounded by postmortem artifacts. The OVX mouse model recapitulates estrogen-deficient OP, but may not fully mimic the complex pathophysiology of age-related OP in humans, including multifactorial influences such as systemic inflammation or comorbidities. Spatial transcriptomic analysis was not performed to validate the precise spatial localization of OP-associated molecular signatures within the hippocampal subregions, limiting insights into regional microcircuit interactions.

## Conclusion

Through multimodal investigations, our findings identify molecular correlates for the brain imaging phenotypes of OP, and highlight astrocytes and synaptic signaling as potential targets for the dual protection of both bone and brain health.

## Data Availability

The original contributions presented in the study are included in the article/**Supplementary Material**. Further inquiries can be directed to the corresponding authors.
