# Supplementary material for: Multimodal integration of neuroimaging, transcriptomics and single-cell analysis reveals molecular correlates linking osteoporosis to brain abnormalities
Source: Front Immunol. 2026 May 18;17:1817475. doi: 10.3389/fimmu.2026.1817475 (PMC13222962; doi:10.3389/fimmu.2026.1817475)
Supplement: Supplementary file 1 [file DataSheet1.docx]

**Functional brain alterations and molecular correlates of cognitive decline in osteoporosis**

**Supplementary Methods**

**Inclusion and exclusion criteria**

In the present study, the diagnostic criteria for osteoporosis (OP) adhered to the guidelines established by the World Health Organization (WHO). Specifically, patients with OP were included if they had a bone mineral density (BMD) T-score of ≤ -2.5, assessed at either the femoral neck or lumbar spine. To ensure diagnostic specificity, we performed thorough biochemical testing to exclude secondary osteoporosis, including measurements of serum calcium, phosphorus, alkaline phosphatase (ALP), parathyroid hormone (PTH), 25-hydroxyvitamin D, and bone turnover markers (e.g., C-terminal telopeptide of type I collagen [CTX] and N-terminal propeptide of type I procollagen [PINP]). A detailed medical history was obtained, including medication use (e.g., glucocorticoids), endocrine disorders, and malignancies, which were reviewed to rule out conditions such as hyperparathyroidism, osteomalacia, or multiple myeloma. Furthermore, exclusion criteria for both OP patients and healthy controls included the following: (1) a history of fracture; (2) comorbidities affecting bone metabolism (e.g., hypercortisolism, hyperthyroidism, glucocorticoid use, and connective tissue disorders); (3) a history of psychiatric or neurological disorders; (4) other complications (e.g., diabetes mellitus, coronary artery disease); (5) alcohol or drug abuse; (6) hormonal medication use; (7) contraindications to MRI scanning; and (8) brain abnormalities (e.g., tumors, cerebral infarctions, hemorrhages, vascular malformations). Participants who met any of these criteria were excluded from the study.

**FMRI data acquisition and preprocessing**

Functional and structural brain MRI data were acquired using a 3T MAGNETOM Prisma MR scanner (Siemens, Erlangen, Germany) and a 32-channel head coil. Resting-state fMRI data were collected from all participants who completed two MRI scans one month apart and were included in the final analysis. During prolonged resting-state fMRI acquisitions, participants with closed eyes tend to experience fatigue or even fall asleep due to repetitive scanner noise, which can compromise the data quality. Thus, participants were asked to keep their eyes open, blink normally, and focus on a dark screen for 10 min, consistent with prior resting-state fMRI acquisition protocols. Functional MRI data were obtained using a gradient-echo echo-planar imaging (EPI) sequence with the following parameters: repetition time (TR) = 2000 ms, echo time (TE) = 30 ms, number of slices = 44, slice thickness = 3.0 mm, in-plane resolution = 2.6 × 2.6 mm, flip angle = 90°, field of view (FOV) = 222 mm × 222 mm, matrix = 72 × 72, slice acceleration factor = 4, phase encoding acceleration factor = 2, bandwidth = 1690 Hz/pixel, and slice orientation = transversal. In total, 300 volumes were acquired. Following functional imaging, a high-resolution T1-weighted structural image was collected using an isotropic voxel magnetization-prepared rapid gradient-echo (MPRAGE) sequence (voxel size = 1 mm³) to enable accurate anatomical localization for downstream analyses. In addition, a 2-inversion contrast magnetization-prepared rapid gradient-echo (MP2RAGE) sequence was obtained with the following parameters: TR/TE = 4000 ms/3.41 ms, inversion times = 700 ms/2110 ms, FA1/FA2 = 4°/5°, matrix = 256 × 256, FOV = 256 mm × 256 mm, number of slices = 192, in-plane resolution = 1 mm × 1 mm, slice thickness = 1.25 mm, sagittal orientation, and total scan duration = 6 minutes and 42 seconds.

fMRI data were preprocessed using the DPARSFA toolbox [19], following a set of systematic procedures to ensure data integrity and reliability. The preprocessing workflow encompassed slice-timing correction, motion realignment to rectify head movement artifacts, and the co-registration of functional scans to each participant’s individual structural images. Subsequently, normalization was conducted to align the images to a standard anatomical template, followed by smoothing with an 8 mm full-width at half-maximum (FWHM) Gaussian kernel to improve the signal-to-noise ratio (SNR). Notably, the smoothing step was performed after the ReHo calculation, with all the other preprocessing steps unchanged. Subsequently, segmentation into gray matter, white matter, and cerebrospinal fluid (CSF) was performed to reduce temporal confounders that might impact subsequent analyses. To isolate relevant neural signals, band-pass filtering was implemented within a frequency range of 0.01–0.1 Hz. To resolve potential correlations arising from head motion and other artifacts, outlier time points in motion parameters and global signal intensity were detected using the Artifact Detection Toolbox (ART, https://www.nitrc.org/projects/artifact_detect). Notably, a time point was classified as an outlier when its composite displacement from the prior image surpassed 0.5 mm or when its global mean intensity differed by over 3 standard deviations (SD) from the average image intensity. These identified outliers, along with motion parameters, were included as regressors in the first-order general linear model (GLM) to account for these confounding influences in subsequent analyses.

**AHBA preprocessing**

Microarray-based transcriptional profiles from the AHBA repository containing postmortem adult brain specimens and preprocessed expression matrices were subjected to hemisphere-specific curation. Based on tissue availability, 1028 left-hemispheric cortical samples were retained. Probe-gene mapping utilized the current NCBI Entrez annotations to ensure the accuracy of the genomic annotations. Following quality assurance protocols, probes with subthreshold detection rates (i.e., intensity below background noise levels in >50% of the samples) were removed. To improve biological validity, RNA-seq concordance filtering was applied to prioritize probes that showed maximal cross-platform correlation for each gene. Normalization uses a robust sigmoid transformation to reduce the platform-specific technical variability across specimens. This multistage curation resulted in standardized transcriptional signatures for 10,185 protein-coding genes per tissue sample, creating a molecular atlas for multimodal integration with neurodynamic parameters. The refined gene-brain mapping matrix was then used to guide spatial correlation analyses with functional neuroimaging biomarkers, supporting mechanistic interpretations of neurophysiological patterns through molecular signatures.

**Neuroimaging-neurotransmitter/metabolism Association Analyses**

The 17 neurotransmitter/metabolic imaging maps selected from the JuSpace toolbox included PET, SPECT, and MRI (arterial spin labeling-derived cerebral blood flow, ASL-CBF) modalities comprising 5-hydroxytryptamine (5-HT) receptor subtypes 1a, 1b, 2a, and 4 (5-HT1a, 5-HT1b, 5-HT2a, 5-HT4), cannabinoid 1 receptor (CB1), cerebral blood flow (CBF), dopamine D1 and D2 receptors (D1, D2), 6-fluoro-(18F)-L-3,4-dihydroxyphenylalanine (FDOPA), dopamine transporter (DAT), noradrenaline transporter (NAT), gamma-aminobutyric acid type A receptor (GABAA), μ-opioid receptor (MU), N-methyl-D-aspartic acid receptor (NMDA), serotonin transporter (SERT), metabotropic glutamate receptor 5 (mGluR5), and vesicular acetylcholine transporter (VAChT). These neurotransmitter/metabolic density maps are widely utilized in existing literature and are based on data from the largest cohort of healthy participants. Covariates, including age, sex, and educational attainment, were adjusted prior to the analysis. Using the JuSpace toolbox with computing option 5 and the neuromorphometrics atlas, Fisher’s z-transformed Spearman correlation coefficients were calculated between the z-scored individual ALFF/ReHo maps (OP patients vs. HCs) and the selected receptor/transporter distribution maps. Subsequently, individual z-transformed maps of patients with OP relative to HCs were generated, and Fisher’s z-transformed Spearman correlation coefficients were computed between these z-scored ALFF maps and the spatial distribution of each selected receptor/transporter density map. A grey matter probability map was used to correct for spatial autocorrelation. Exact p-values were determined via 10,000 permutations, with one-sample t-tests used to verify whether the observed correlation coefficients differed significantly from zero. Multiple comparisons were corrected using the false discovery rate (FDR), with statistical significance set at p < 0.05.

**snRNA-seq data analyses**

To achieve a balanced nuclear representation across different brain regions, we employed the sample function to randomly select 25% of the total nuclei from each sample. Subsequently, quality control (QC) was conducted on the combined dataset and nuclei with fewer than 800 unique molecular identifier (UMI) counts were removed. To address the batch effects originating from distinct studies, batch correction was performed using the Harmony package (v1.0; <https://github.com/immunogenomics/harmony>). Harmony integrates datasets into a common low-dimensional space, effectively reducing batch effects while retaining the inherent biological variability. Dimensionality reduction, clustering, and cell-type annotation were then performed using the Seurat R package (v4.0.0; <https://satijalab.org/seurat/>).

Single-nucleus RNA sequencing (snRNA-seq) data from the human brain were acquired in this study. Transcriptomic datasets were retrieved from Siletti et al. (2023) and the CELLxGENE repository (https://cellxgene.cziscience.com). To analyze the snRNA-seq data, we first combined datasets from all brain regions into a single unified dataset (Figure S1). Preprocessing steps could be found in supplementary methods-Single-nucleus RNA sequencing (snRNA-seq) data preprocessing. The cell types were annotated based on previously published canonical marker genes. For instance, astrocytes were identified by the marker genes AQP4, GFAP, and GJA1; Schwann cells by MPZ and S100B; oligodendrocytes by MOG and MOBP; and oligodendrocyte precursor cells (OPCs) by VCAN. Other cell populations such as CNS macrophages, leukocytes, fibroblasts, and neurons were annotated using well-validated marker genes in the same manner.

To assess the brain region specificity of the gene set identified from neuroimaging-transcriptome association analyses, we used the Cell-type-specific Expression (CELLEX) algorithm (Python module) to compute region-specific expression profiles from the snRNA-seq data [14]. The analytical workflow is illustrated in Figure 1. CELLEX combines differential expression T-statistics, gene enrichment scores, and expression proportions to measure region-specific gene expression levels. We then conducted an enrichment analysis by intersecting the significantly associated gene sets with region-specific expression profiles. A hypergeometric test was applied to evaluate the statistical significance of gene enrichment in each brain region, with FDR < 0.05, which was used as the cut-off for defining brain regions enriched with the target gene set. Annotations for these enriched brain regions were enhanced by referring to previously published canonical brain region markers and anatomical classification.

Non-negative matrix factorization (NMF) was implemented on the batch-effect-corrected and standardized snRNA-seq expression matrix (via the NMF R package v0.23.0) to break down the transcriptomic data into latent gene programs. The optimal number of gene programs was ascertained using cophenetic correlation coefficients and silhouette scores to guarantee biological significance. Genes within each program were sorted according to their contribution weights and the top 200 high-weight genes per program were chosen for functional annotation. Functional enrichment analysis was carried out using the Kyoto Encyclopedia of Genes and Genomes (KEGG) and Gene Ontology (GO) databases (concentrating on biological processes), with FDR < 0.05 regarded as statistically significant. This step sought to characterize the biological pathways and functional roles of each identified gene.

Finally, we integrated two gene subsets to define the core gene set for protein-protein interaction (PPI) analysis: (1) the neuroimaging-transcriptome associated gene set (generated in the previous neuroimaging-transcriptome association analysis) and (2) the top 200 high-weight genes from the identified gene program. The intersection gene set was uploaded to the STRING database (v11.5, https://string-db.org/), a comprehensive resource for predicting and annotating protein-protein interactions, with Homo sapiens set as the target species. To filter high-confidence interactions, we applied a medium confidence threshold (combined score ≥ 0.4), which is a widely accepted cutoff for balancing sensitivity and specificity in PPI network construction. To explore the biological relevance of the PPI network, we first identified the functional modules (clusters) within the network using the MCODE plugin (v2.0.0) in Cytoscape. MCODE uses a density-based algorithm to detect tightly connected sub-graphs. Each identified module was then subjected to functional enrichment analysis using the clusterProfiler R package (v4.6.0), with enrichment against the KEGG pathway and GO biological process databases. Statistical significance was defined as FDR < 0.05, and only enriched terms with ≥ 3 genes were retained to ensure biological significance.

**Animal Study**

Ten- to 12-week-old female C57BL/6J mice (n=60) were acquired from ShuLaiBao Biotechnology Co. Ltd. (Hubei, China). All animals were housed in a specific pathogen-free (SPF) facility at the Xi’an Honghui Hospital. The mice were housed in groups and maintained under controlled conditions: temperature of 20–24 °C, humidity of 40–60%, and a 12-h light/12-h dark cycle. The animals were provided with a standard laboratory chow diet and had unrestricted access to food and water. Prior to surgery, all mice were anesthetized using isoflurane and the fur over the surgical site was clipped. A 1-cm incision was gently made in the skin and peritoneum, approximately 1 cm lateral to the spine. In the ovariectomized (OVX) model group, bilateral ovaries were resected and ligated; in contrast, the control group underwent excision of a small fat pad without ovarian removal. The incision was then carefully sutured and povidone-iodine was used for disinfection. Euthanasia was conducted via CO₂ inhalation, followed by cervical dislocation to ensure death.

All behavioral assessments were conducted during the light phase of the 12-h light/12-h dark cycle in a sound-attenuated, dimly lit room with illumination provided by wall lamps placed in the corners. Prior to each test, all mice were allowed a 30-minute acclimation period to the testing environment. Behavioral evaluations were scheduled between 08:30 h and 12:30 h, with consistent scheduling across the experimental groups. All assessments were performed by experimenters who were blinded to the treatment groups. Spatial learning and memory were evaluated using the Morris Water Maze (MWM) test, administered 3 months after ovariectomy (OVX) surgery, in accordance with a previously established protocol (details reported in [1]). Before the formal test, the mice were pre-trained to locate a submerged transparent platform (10 × 10 cm) within a white circular pool (120 cm diameter, 22 cm height) filled with water at room temperature. During pre-training, the mice were restricted to a limited swimming zone (70 cm × 13 cm). Three days after pre-training, the MWM test commenced, encompassing two phases: acquisition and probing. The acquisition phase consisted of five daily acquisition trials for five consecutive days, with each trial initiated from one of four randomly selected positions around the pool. For each trial, a mouse was placed in the pool facing the wall, and the trial was terminated when the mouse reached the escape platform or after 60 s if the platform remained undetected. On the fifth day of the acquisition phase, a probe trial was performed in which the escape platform was removed. Each mouse was placed in the pool for 60 s, and their search behavior was recorded to evaluate search preference, which is a measure of spatial memory. Between consecutive trials, the mice were allowed a 5-minute rest period in a heated cage to maintain body temperature. Swimming trajectories of each mouse were captured using an overhead video camera, and behavioral parameters were quantified using EthoVision XT 7.1 video tracking software (Noldus Information Technologies, The Netherlands). For the acquisition phase, the analyzed parameters included swimming speed (cm/s), escape latency (s), and time spent in the wall zone (defined as the area within 15 cm of the pool wall). For the probe trial, additional metrics included the latency to enter the target zone (the 30-cm radius area where the platform had been located) and the total distance traveled within this zone.

For micro-CT analysis, fixed non-demineralized mouse femurs were scanned using a Bruker μCT system (Skyscan 1276 Micro-CT, Kontich, Belgium). Scan parameters were set as follows: medium resolution with a 6.5 μm voxel size, 70 kV voltage, 200 mA current, a 0.25 mm aluminum filter, and an integration time of 350 ms. Three-dimensional (3D) images were created from contoured two-dimensional (2D) images via distance transformation-based techniques applied to the original grayscale images (CTvox, version 3.3.0). Both 3D and 2D analyses were performed using CT Analyzer software (version 1.18.8.0). Bone microarchitecture was evaluated within a 1.5 mm-long region of interest (ROI) that started 0.5 mm proximal to the distal growth plate for trabecular bone measurements.

For Western blotting, protein lysates from bone marrow stromal cells (BMSCs) and bone marrow macrophages (BMMs) were prepared using RIPA buffer (Thermo Fisher Scientific, USA). Samples were resolved using 10% SDS-PAGE and transferred to polyvinylidene fluoride (PVDF) membranes (Millipore, Billerica, MA, USA). Wet transfer was used in all assays. Electrophoresis was conducted at a constant voltage of 80 V for 20 min followed by 120 V for 60 min. For protein transfer, the membranes were incubated at a constant current of 250 mA for 80 min. Membranes were blocked with 5% bovine serum albumin (BSA) diluted in TBST and incubated overnight at 4 °C with primary antibodies against GFAP (Sigma-Aldrich, SAB5201124), RGS7 (ThermoFisher, PA5-118241), and RGS6 (ThermoFisher, PA1-41407). Sample loading was performed using 20 μg protein per lane.

For histological and immunohistochemical examinations, mouse tibiae and femurs were fixed and decalcified in EDTA solution (Aladdin, China) for 10 days. After dehydration and paraffin embedding, 6 μm-thick tissue sections were generated. These sections were subjected to xylene dewaxing and ethanol rehydration sequentially, followed by staining with primary antibodies against osteocalcin (Servicebio, GB120012-100), TRAP (Servicebio, G1050-50T), and GFAP (Sigma-Aldrich, SAB5201124), according to standard protocols. Subsequently, sections were dehydrated, cleared, and mounted.

Morphological analysis of GFAP-positive astrocytes was conducted on images acquired from the same brain sections used for GFAP staining. For this analysis, the hippocampal region from one 20 μm sagittal section per mouse was imaged. Images were captured at 40× magnification using a Zeiss Axio Imager M2 equipped with a digital camera (Axiocam, Zeiss, Germany) and the ZEN software. Individual images were stitched to eliminate overlap between adjacent tiles and exported in TIFF format. The count of GFAP-positive cells and quantitative analysis of cellular morphological parameters were performed via digital modeling using the MicroTrac analysis platform [2]. Cells that could not be correctly reconstructed using the software were manually excluded from the analysis.

**References**

[1] R. Minkeviciene, P. Banerjee, H. Tanila, Cognition-enhancing and anxiolytic effects of memantine, Neuropharmacology 54(7) (2008) 1079-85.

[2] M. Abdolhoseini, F. Walker, S. Johnson, Automated tracing of microglia using multilevel thresholding and minimum spanning trees, Annu Int Conf IEEE Eng Med Biol Soc 2016 (2016) 1208-1211.

**Table S1. Demographic Data**

|  | OP | Control | p-value |
| --- | --- | --- | --- |
| Age | 61.5±6.7 | 62.1±7.2 | 0.67 |
| Sex | 26/26 | 26/26 | 1 |
| Years of education | 7.8±4.5 | 7.6±5.2 | 0.83 |
| Time since osteoporosis diagnosed (years) | 5.6±4.7 | NA |  |
| MoCA scores | 19.6±2.8 | 25.3±3.7 | <0.0001 |
| BMD | -2.9±0.29 | -1.7±0.31 | <0.0001 |
| Mean FD Jenkinson | 0.05±0.02 | 0.05±0.03 | 0.95 |

The demographic and clinical assessment of osteoporosis (OP) and healthy controls. MoCA: Montreal Cognitive Assessment, BMD: Bone mineral density, FD: Framewise displacement.

**Figure S1**

**
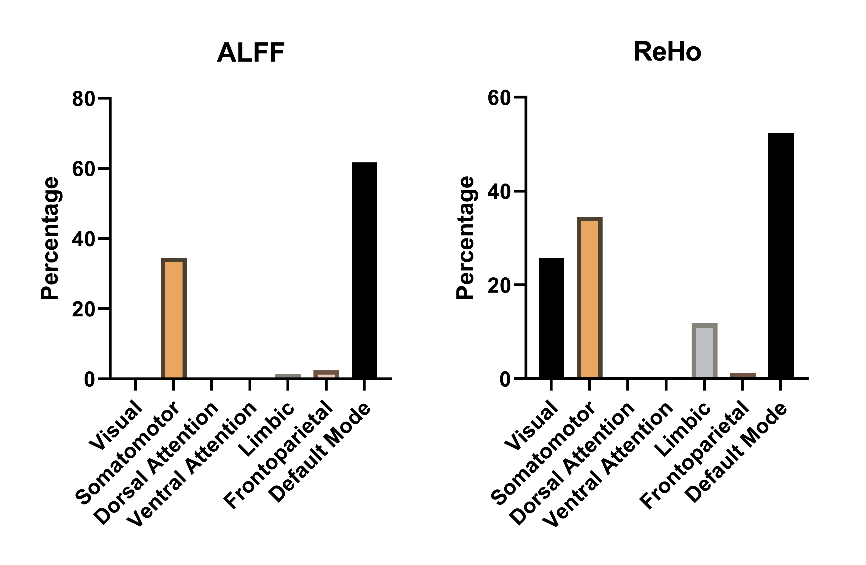
**

The percentage was defined as the number of vertices showing significant between-group differences that overlapped with a specific network from the Yeo 2011 7-network atlas, divided by the total number of vertices showing significant between-group differences.

**Figure S1.**

**
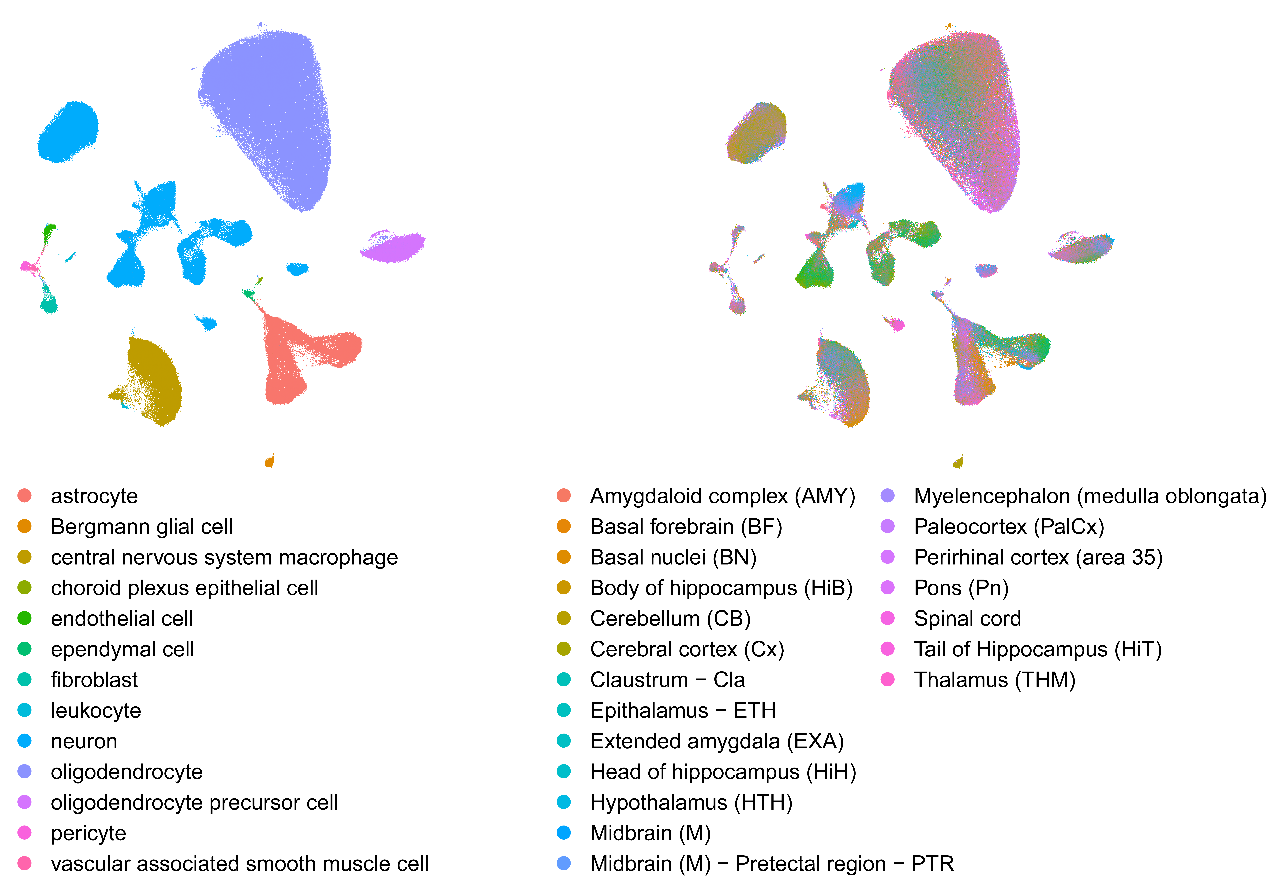
**

Left Panel. Cell types are differentiated by colors, covering various central nervous system cell types including astrocytes, neurons, oligodendrocytes, etc. Right Panel. Colors correspond to the brain region tissue origins of the cells, involving multiple brain structures such as the Amygdaloid complex, Basal nuclei, Cerebral cortex, and distinct regions of the hippocampus.

**Figure S2.**

**
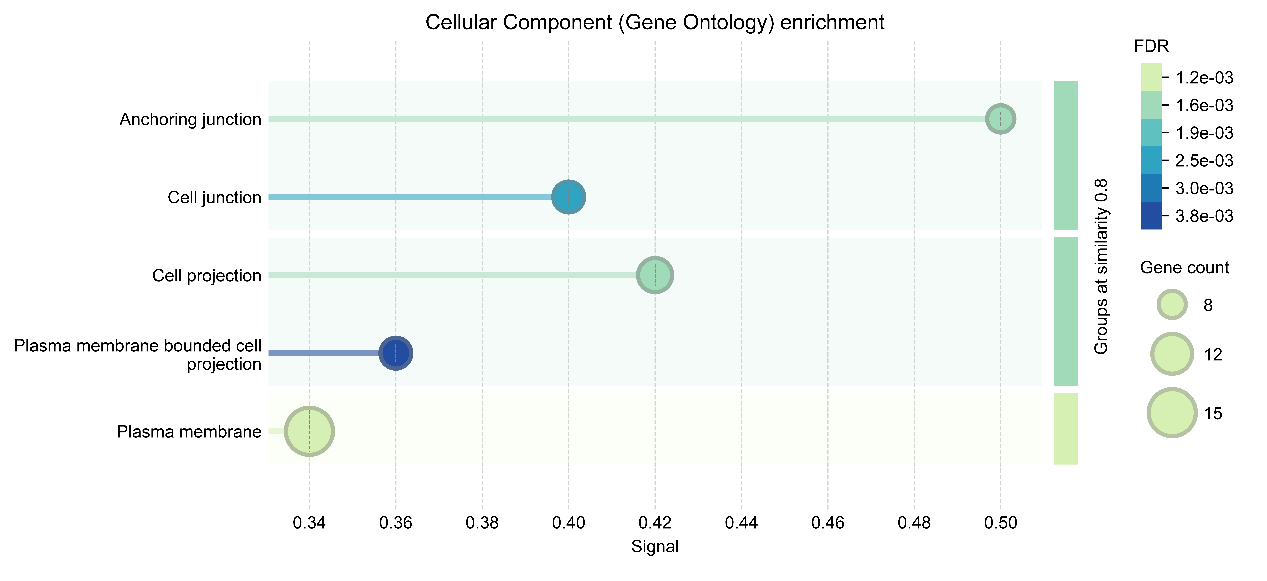
**

Cellular Component enrichment analysis of Gene Ontology (GO) terms using genes for PPI analysis.
